# Graves’ Masquerade: A Case of Resistance to Thyroid Hormone (RTH) Syndrome

**DOI:** 10.7759/cureus.78160

**Published:** 2025-01-28

**Authors:** Yik Hin Chin, Dorothy Maria Anthony Bernard, Siew Hui Foo

**Affiliations:** 1 Endocrinology, Diabetes, and Metabolism, Hospital Selayang, Selangor, MYS; 2 Endocrinology, Diabetes, and Metabolism, Hospital Selayang, Selayang, MYS

**Keywords:** graves, graves' disease, resistance to thyroid hormone, rth, tft, trh stimulation test, tsh-oma

## Abstract

Resistance to thyroid hormone (RTH) syndrome is characterized by reduced sensitivity to thyroid hormones (TH). It is an autosomal dominant genetic disease commonly caused by a mutation of the thyroid hormone receptor beta (THR-β) gene. Manifestations of RTH can be variable, as one can be clinically euthyroid to hyperthyroid or hypothyroid. We would like to share a case of resistance to thyroid hormone beta (RTH β) that presented with atrial fibrillation (AF) and was treated as Graves' disease with antithyroid drugs initially but was otherwise clinically euthyroid. The diagnosis was subsequently revised to RTH after a delay of more than eight years when the patient was referred to endocrinology for an aberrant thyroid function test with persistently raised TH and an unsuppressed thyrotropin level after performing the appropriate investigations. This case illustrates the challenge in diagnosing RTH in individuals with apparently preserved cardiac tissue TH sensitivity mimicking Graves' disease and the importance of recognition of RTH to avoid inappropriate therapy to suppress the compensatory increase in TH production to maintain the euthyroid state in affected individuals.

## Introduction

Resistance to thyroid hormone (RTH) syndrome is characterized by reduced sensitivity to thyroid hormones (TH). It is an autosomal dominant genetic disease commonly caused by a mutation of the thyroid hormone receptor beta (THR-β) gene. This clinical syndrome was first described in 1967 [1]. It is a syndrome manifested by reduced sensitivity to THs. Its prevalence varied according to sources; however, it was believed to be seen in one in 40,000 of the population and one in 19,000 live births, respectively. Manifestations of RTH can be variable, as one can be clinically euthyroid to hyperthyroid or hypothyroid. Resistance to thyroid hormone syndrome usually presents with elevated serum levels of free thyroxine (FT4) and free triiodothyronine (FT3) accompanied by a normal or slightly elevated serum thyroid-stimulating hormone (TSH) level [2]. We report a case of a patient with resistance to thyroid hormone beta (RTH-β) who appeared to have selectively preserved cardiac tissue sensitivity to TH but was otherwise euthyroid metabolically.

## Case presentation

The patient was a 47-year-old Chinese male who first presented to Hospital Selayang in Selangor, Malaysia, in December 2015 at the age of 37 years old with heart failure in atrial fibrillation (AF). There was no other known cardiovascular risk factor then except being obese with a body mass index (BMI) of 33.3 kg/m² associated with dyslipidemia and gout. Thyroid function test (TFT) showed a high FT4 of 28 pmol/L (11.5 - 22.7) with normal TSH of 1.3 pmol/L (0.38 - 5.33). Thyroglobulin (anti-TG) and thyroperoxidase (anti-TPO) antibodies were negative. He was managed as a case of Graves’ disease and commenced on oral carbimazole 10 mg daily on top of warfarin and medical therapy for heart failure. He was documented to be euthyroid clinically otherwise throughout. There was no known family history of thyroid disorders.

After a total of 17 months, oral carbimazole was stopped as the TFT apparently normalized on a single occasion, although the serial FT4 prior to that did not show any significant change (Table 1). He remained stable at New York Heart Association (NYHA) Class 1 while subsequent TFTs yielded similar results of high FT4 with a normal TSH in the absence of symptoms of hyper- or hypothyroidism throughout.

**Table 1:** Serial thyroid function tests of the patient from 2016 till 2024 with abnormal values in bold Reference ranges: ^a^ FT4 11.5-22.7 pmol/L; FT3 3.5-6.5 pmol/L; TSH 0.35-5.50 uIU/mL; ^b^FT4 7.86-14.41 pmol/L; FT3 3.8-6.0 pmol/L; TSH 0.38-5.33 uIU/mL; ^c^FT4 9.0-25.0 pmol/L; FT3 3.5-6.5 pmol/L; TSH 0.40-4.70 uIU/mL *Reference ranges of a, b, and c depict the differences in TFT range upon using different TFT platforms. The hospital used a different TFT platform through the years. Jan 2016 to Jul 2020: The hospital used reference range (a) August 2021-present; The hospital used reference range (b) due to a change of vendor and machine; March 2024: TFT was sent to another laboratory (c) out of the hospital to rule out assay interference, which was an important differential diagnosis. FT4: free thyroxine; FT3: free triiodothyronine; TSH: thyroid stimulating hormone; TFT: thyroid function test

	Jan 2016 ^a^	May 2017 ^a^	Nov 2019 ^a^	Jul 2020^ a^	Aug 2021 ^b^	Dec 2023 ^b^	Mar 2024 ^b^	Mar 2024 ^c^
FT4 (pmol/L)	28	Normal	23.7	27.5	20.8	23.2	25.1	36.7
FT3 (pmol/L)							8.2	12.5
TSH (uIU/mL)	1.3	Normal	2.33	1.51	1.85	1.80	1.47	1.74
Carbimazole (mg / day)	10	stopped	-	-			-	-

In November 2023, the patient was admitted for non-ST elevation myocardial infarction and was discharged with double antiplatelet therapy on top of warfarin. This was followed by the development of lower rectal bleeding shortly after home discharge. Colonoscopy showed a large rectosigmoid colon tumor about 20 cm from the anal verge, occupying 25% of the colonic lumen. Histopathological examination of the mass was consistent with rectosigmoid adenocarcinoma. A coronary angiogram performed in January 2024 revealed a 90% left anterior descending artery (LAD) stenosis, and a drug-eluting stent was inserted. As the computed tomography of the thorax, abdominal, and pelvis (CT-TAP) showed the mass was confined to the colon with no distant metastasis, he was planned for anterior resection three months after for coronary revascularization.

Referral to endocrinology was made for further evaluation and management of the abnormal TFT during the preoperative assessment. Upon assessment, the patient was clinically euthyroid with no palpable goiter. Thyroid autoantibodies, including TSH receptor antibody (TRAB), remained negative. Repeated TFT in another laboratory using a different assay platform showed a similar TFT pattern. In view of the persistently raised FT4 with a normal TSH since 2015, associated with a predominantly euthyroid clinical state, an initial impression of RTH secondary to THR-β mutation (RTH- β) was made. A collective decision was made for him to proceed with anterior resection for rectosigmoid adenocarcinoma under general anesthesia without any specific thyroid treatment except for perioperative beta blockers.

**Figure 1:**
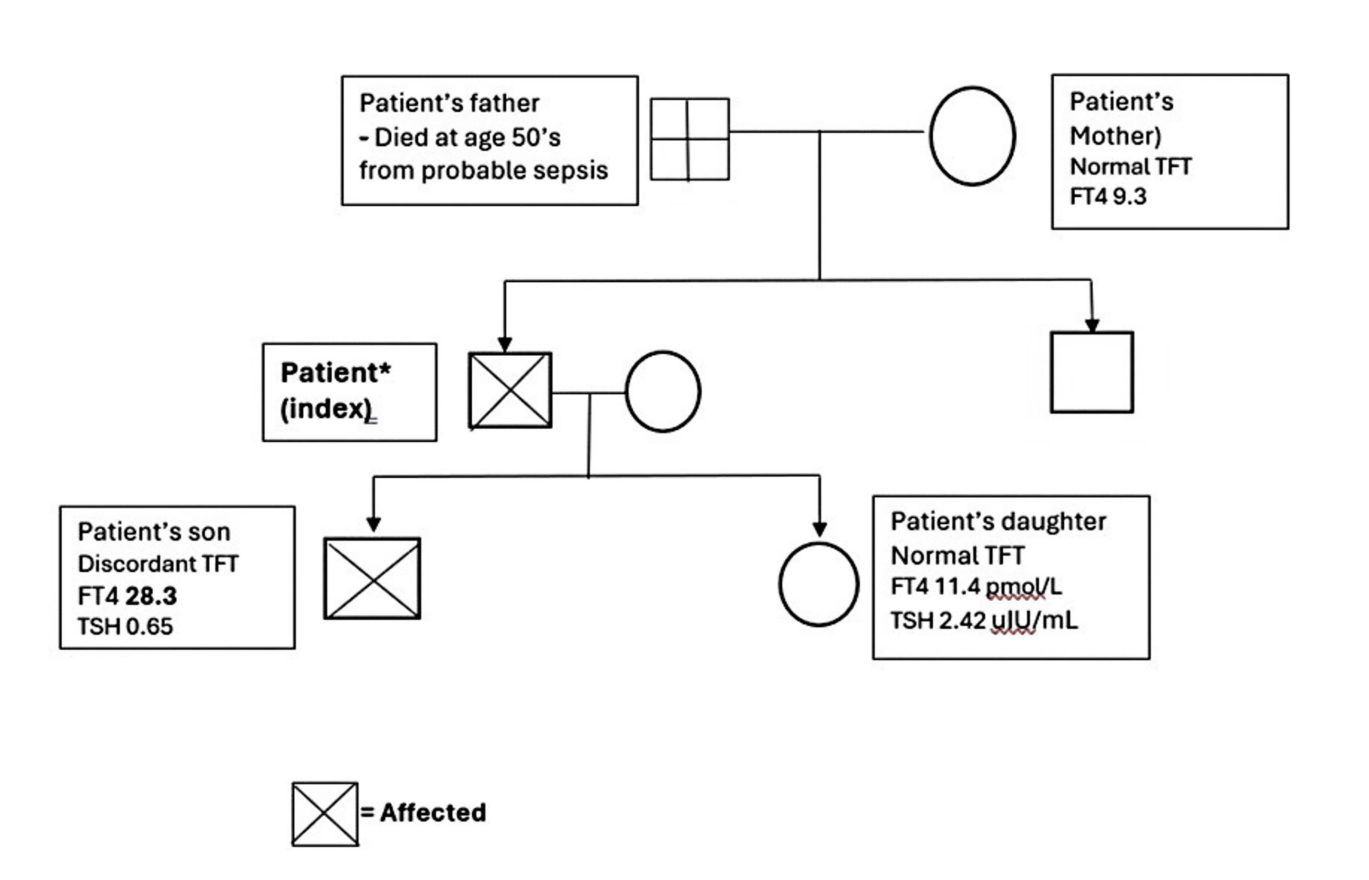
Family pedigree of the patient and their thyroid function test results FT4: free thyroxine (unit: pmol/L); TSH: thyroid stimulating hormone (unit: uIU/ml); TFT: thyroid function test

He underwent the surgery in April 2024. The surgery was uneventful, and he was discharged well on day five postoperatively. The TFTs performed on his other first-degree family members revealed a similar pattern of discordant TFT in his son, who was also clinically euthyroid (Figure 1). Further investigations performed revealed a normal sex hormone-binding globulin (SHBG), a mildly elevated alpha-subunit, an exaggerated TSH in response to thyrotrophin stimulation, and the absence of a pituitary adenoma on magnetic resonance imaging (MRI) (Table 2). A genetic study for the mutational defect in the THR-β gene was not available. The final diagnosis was RTH-β.

**Table 2:** Biochemical investigations performed for patient SHBG: sex hormone-binding globulin; Anti-Tg: anti-thyroglobulin; Anti-TPO: anti-thyroperoxidase; TRAB: TSH receptor antibody; TRH: thyroid-releasing hormone; TSH: thyroid-stimulating hormone

Test		Results	Reference range / Normal response
Alpha-subunit (IU/L)		1.04	0 to 0.7
SHBG (nmol/L)		39.2	13.3- 89.5
Thyroid autoantibodies	Anti-Tg /Anti-TPO	Negative	<1.75
	TRAB (iu/L)	<0.8	
TRH stimulation test (µIU/mL)		0 min = TSH 1.42	TSH rises to >5 µIU/mL with its 30-minute exceeding the 60-minute value
		30 min = TSH 11.11	
		60 min = TSH 7.23	

## Discussion

Thyroid hormones function as a key regulator in human health, which includes brain development, tissue differentiation, bone growth, cardiovascular homeostasis, and glycolipid metabolism. Its balance is mediated by a negative feedback loop in the body via the hypothalamic-pituitary-thyroid (HPT) axis. The balance and circulation of THs can be interrupted in several physiological steps, including defects in receptor-dependent transactivation, eventually leading to RTH, which is mainly caused by mutational defects in the THR-β [3], encoded by THR-β genes on chromosome 3. The clinical manifestation of RTH-β varies depending on the location and type of genetic mutation, making the disease phenotypically heterogeneous.

Resistance to thyroid hormone beta is generally divided into three categories: general RTH-β, pituitary RTH-β, and peripheral RTH-β [4]. This has been used to describe different clinical manifestations of RTH-β, suggesting tissue variability in the resistance to thyroid hormone. The term generalized resistance to thyroid hormone (GRTH) was applied to most patients with RTH-β that were able to maintain a euthyroid state usually. On the other hand, pituitary resistance to thyroid hormone (PRTH) refers to patients with RTH-β who have symptoms of thyroid excess in peripheral tissues or demonstrated changes in peripheral tissue markers compatible with thyroid hormone action without significant suppression of TSH [5].

The main differential diagnoses of elevated TH with unsuppressed TSH in patients include RTH-β versus TSH-producing pituitary adenoma (TSH-Oma) [6]. The patient had clinical features and a natural history consistent with RTH-β. Laboratory assay interference was unlikely in view of the consistently raised TH with unsuppressed TSH on different assay platforms. RTH-β usually presents in a euthyroid state similar to him. On the contrary, a person with TSH-Oma is usually hyperthyroid clinically. A family history of autosomal dominant inheritance was present in 85% of RTH-β [2], while the presence of a pituitary macroadenoma was observed in 75% of TSH-Oma [7]. 

**Figure 2:**
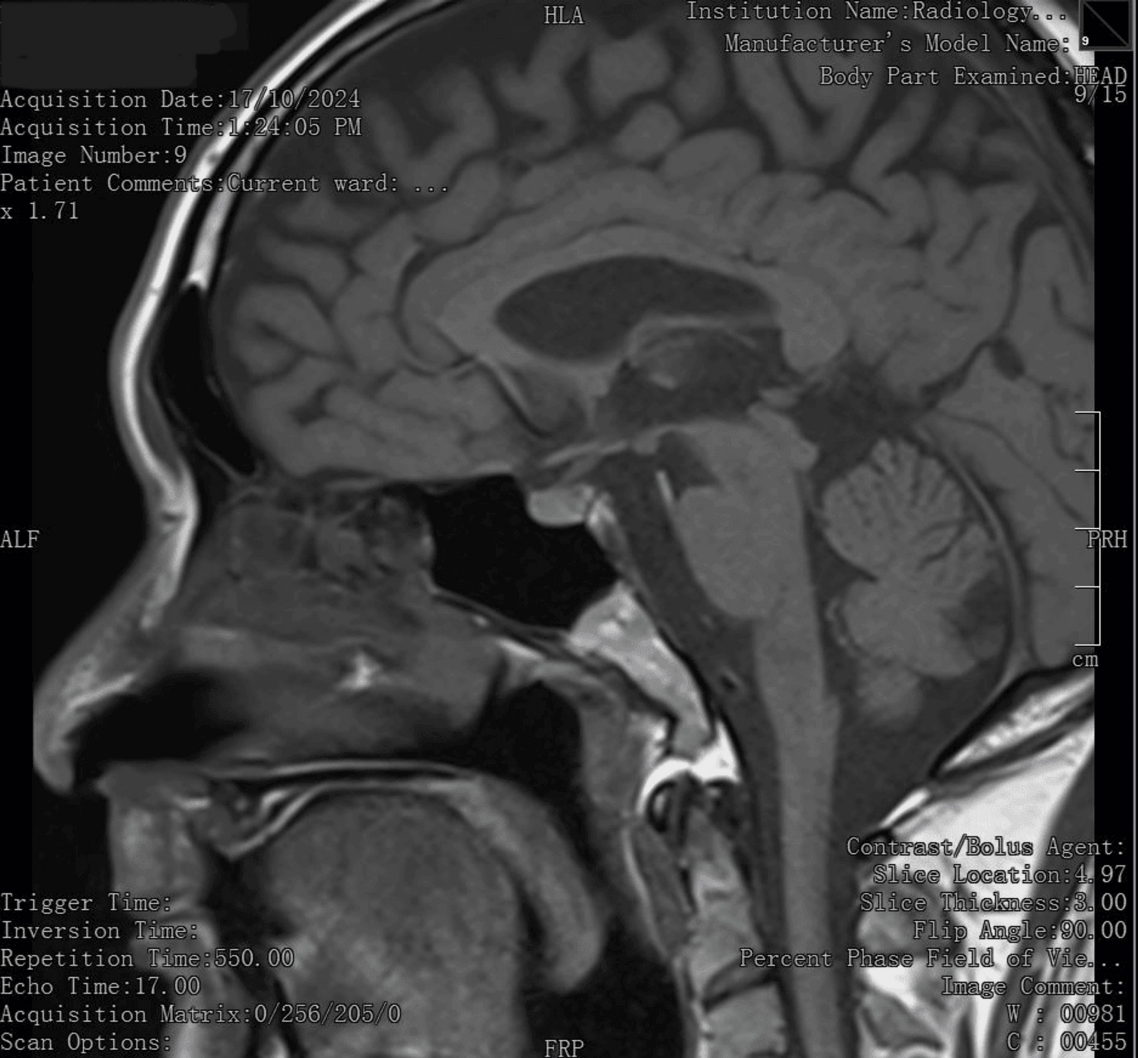
Magnetic resonance imaging of the brain (sagittal view) depicted no lesions at the pituitary.

As genetic screening was not assessable in our setting, the diagnosis of RTH-β was made based on the clinical presentation, indolent natural history, biochemical findings, absence of pituitary adenoma on MRI (Figure 2), and an apparent autosomal dominant inheritance pattern in the family pedigree. The exaggerated TSH response on the TRH stimulation test and normal SHBG was consistent with RTH-β. The modestly raised alpha subunit was the only biochemical finding inconsistent with RTH; TSH-producing pituitary adenoma was deemed unlikely in the absence of any discernible lesion on the MRI pituitary at close to 10 years after the initial presentation.

Even though the patient was relatively spared from the manifestations of thyrotoxicosis in the peripheral tissue, he had an early onset of AF complicated by heart failure. Resistance to thyroid hormone beta often had elevated TH levels to compensate for the peripheral tissue resistance to TH, to maintain a euthyroid metabolic state. However, the high endogenous TH levels sometimes can exert hyperthyroid effects, particularly in tissues that express predominantly thyroid hormone receptor alpha (TR-α), the other isoform of THR, such as the heart and bone [8]. These tissues do not express much THR-β, which is predominantly found in the hypothalamus-pituitary, liver, kidney, and lung. Therefore, it is possible that the preserved sensitivity to THR-α was responsible for the early cardiovascular manifestations in our patient. Similarly, a case reported by Lai et al. showed a middle-aged gentleman who had a mutation in the THR-b gene presented with AF and heart failure symptoms but was otherwise clinically euthyroid. Further investigation also showed that his son had the same THR-β gene mutation [9].

Thus, the clinical spectrum in RTH-β is quite broad and overlapping, even among carriers of the same THR-β mutation and within the same family, suggesting that the classifications of generalized and pituitary RTH-β are rather semantics to describe a varying range of clinical signs and symptoms resulting from altered sensitivity to thyroid hormone in different tissue within the same individual [10].

Treatment for RTH should be individualized based on a person’s symptoms and clinical picture instead of attempting to normalize the elevated thyroid hormone levels [11]. Most patients could adequately overcome the TH resistance by increased TH secretion and therefore do not require TH administration unless in the setting of limited thyroid reserve [11]. A person with hypothyroid or hyperthyroid symptoms may require TH, beta-blockers, or TH analogs [6]. In our patient, an attempt to reduce the elevated TH with antithyroid medication may lead to a hypothyroid state prior to a major colorectal surgery under general anesthesia, leading to an increased risk of adverse events such as failed extubation, pulmonary aspiration, etc.

## Conclusions

Resistance to thyroid hormone beta remains a clinically important condition that should not be missed or misdiagnosed. As genetic testing remains out of reach to general masses, diagnosing RTH-β remains a clinical challenge, as most patients are asymptomatic of peripheral manifestations of thyrotoxicosis throughout. As described, the patient was initially treated for Graves' disease and given antithyroid medication, which did not benefit him. Therefore, early recognition and accurate diagnosis of RTH help to avoid inappropriate or unnecessary treatment, such as anti-thyroid drugs or thyroid ablative therapy, that will lead to the need for supraphysiological doses of TH replacement, which may worsen individuals with preserved sensitivity in tissue with predominant expression of THR-α. The patient remained asymptomatic and clinically euthyroid in each clinic visit without medications.
